# Virtuous Organizational Practices: A New Construct and a New Inventory

**DOI:** 10.3389/fpsyg.2021.724956

**Published:** 2021-10-13

**Authors:** Julia Aubouin-Bonnaventure, Evelyne Fouquereau, Hélène Coillot, Fadi Joseph Lahiani, Séverine Chevalier

**Affiliations:** ^1^QualiPsy EE 1901, Psychology Department, University of Tours, Tours, France; ^2^AD CONSEIL, Malakoff, France

**Keywords:** organizational virtuousness, virtuous organizational practices, virtuous organizational practices inventory, VOPi, scale, psychological health, well-being

## Abstract

Research on the identification of organizational practices that promote individual and organizational performance is now very extensive. However, several studies have revealed the dark side of these practices on employees’ psychological health. Consequently, researchers have called on the scientific community to focus on the well-being of workers and to identify the organizational practices that promote it. Thus, the aim of the present research was to fill this gap by introducing a new conceptualization of organizational practices supporting the psychological health of employees and proposing a new improved measure to assess them. Drawing on the American Psychological Association’s model of *Psychologically healthy workplace*, we first conceptualized the innovative multidimensional construct of virtuous organizational practices. We then conducted four studies (*N* = 1,407) to develop and validate the *Virtuous Organizational Practices inventory*. Results of exploratory statistical analyses provide strong evidence of the second-order factor structure of the inventory in different French samples and of the convergent, predictive and incremental validity of this tool. Implications for researchers, organizations and practitioners and avenues for future research are discussed.

## Introduction

Since the 1990s, research on organizational practices that promote workers’ performance and commitment has aroused growing interest in the scientific community (Wang et al., 2020). For example, Murphy et al. (2018) recently carried out a literature review on this subject and identified a preliminary database of nearly 16,000 publications between 1991 and 2015. This vast field of scientific research currently includes many constructs (e.g., *High-performance work system*, *High-involvement work system*, *High-commitment work system*, *Human resources practices*, and *Strategic human resources management*). Because the practices are chosen on empirical rather than conceptual grounds, researchers have difficulty agreeing on the number and nature of practices to be considered (Mendelson et al., 2011; Boon et al., 2019). Nevertheless, numerous studies have shown that these organizational practices are related to improved individual performance, including task performance and organizational citizenship behavior (e.g., Boon et al., 2011; Zhang et al., 2018) and to organizational efficiency, including work unit productivity, financial outcomes (e.g., sales growth, return on invested capital), product/service quality and customer satisfaction, and also to a decrease in labor costs, absenteeism, voluntary turnover, and hospital mortality rates (Combs et al., 2006; West et al., 2006; Guthrie et al., 2009; Jiang et al., 2012; Raineri, 2017; Saridakis et al., 2017; Schmidt and Pohler, 2018).

As underlined by some authors, the effects of these organizational practices on workers’ psychological health are only a secondary concern (Guest, 2017), the employees’ psychological health being considered only as a means to optimize their performance. Moreover, while several studies have shown that these organizational practices have positive relationships with some positive outcomes in terms of workers’ health (e.g., job satisfaction, affective commitment: Kooij et al., 2010; Domínguez-Falcón et al., 2016), some authors observe that it is too early to consider that these necessarily improve well-being (Zhang et al., 2013). Indeed, some research has revealed the “dark side” of these organizational practices (Han et al., 2020); for example, several studies have concluded that they degrade employees’ working conditions by increasing job demands, such as heavier workloads and more demands of service quality (Ramsay et al., 2000; Jensen et al., 2013; Avgoustaki, 2015; Oppenauer and Van De Voorde, 2016; Wang et al., 2019). Kroon et al. (2009) found a positive relationship between high-performance work and burnout, which was fully mediated by job demands, and Oppenauer and Van De Voorde (2016) observed that these practices were positively linked to work overload, which was significantly related to emotional exhaustion. Other studies have underlined the direct harmful effects of these organizational practices on workers’ psychological health, such as an increase in work-life interference, anxiety, perceived stress, burnout and turnover intentions (Godard, 2001; Wood and de Menezes, 2011; Jensen et al., 2013; Topcic et al., 2016).

According to some researchers, these results indicate that organizational practices leading to increased performance are not necessarily the same as those leading to employees’ psychological well-being (e.g., Guest, 2017; Loon et al., 2018). Consequently, they stress the importance of identifying organizational practices that promote employees’ health. Some work in this direction has recently emerged (Guest, 2017; Cooper et al., 2019; Huettermann and Bruch, 2019; Salas-Vallina et al., 2021), but with several important limitations. First, there is significant conceptual confusion in this research area, notably between formal and informal practices, or organizational and managerial practices (Guest, 2017; Cooper et al., 2019; Salas-Vallina et al., 2021). Secondly, most studies have examined these practices from the perspective of senior executives and HR actors (Huettermann and Bruch, 2019), while it has been clearly demonstrated that the workers’ perception of organizational practices is the best predictor of their health, attitudes and behaviors at work (Edgar and Geare, 2005). Thirdly, while the measured constructs have been given new names (e.g., wellbeing-oriented human resource management practices, health-related HRM), the tools used to assess them are the same as those used to measure organizational practices propitious to individual performance (Cooper et al., 2019). In other words, to our knowledge, there is currently no specific validated scale measuring workers’ perceptions of formal organizational practices that promote their psychological health. Finally, although there are numerous studies about organizational practices that promote employees’ well-being, they over-simplify these practices because they do not measure their combined effect (Kooij et al., 2010; Butts et al., 2013). Thus, no integrative psychological construct currently exists in this research domain, and there is a real lack of accurate measures to improve the understanding of the effects of organizational practices specifically focused on workers’ well-being and professional quality of life.

### The Present Research

First, this research aimed to develop a new integrative psychological construct of organizational practices promoting employees’ psychological well-being. Based on the concept of organizational virtuousness and the model of *Psychologically healthy workplace* developed by the American Psychological Association (APA), this paper presents the construct of Virtuous Organizational Practices (VOP). The second aim was to create and validate a new scale (i.e., Virtuous Organizational Practices inventory, VOPi) to assess these practices. To this end, we conducted four studies involving a total of 1,407 French workers from various sectors (e.g., private, public, and non-profit organizations). The purpose of the first study was to generate a pool of items constituting the framework of the VOPi for subsequent testing. The second and third studies tested the VOPi’s factor structure through exploratory and confirmatory factor analyses. In the fourth study, we tested the predictive, convergent and incremental validity of the VOPi.

### Virtuous Organizational Practices

As mentioned above, the first aim of the present study was to contribute to research on the organizational practices promoting workers’ well-being by developing a new integrative conceptual approach, based on the concept of organizational virtuousness derived from positive psychology and the APA model of *Psychologically healthy workplace.* The concept of organizational virtuousness (Meyer, 2018) emerged from the field of positive organizational scholarship. Broadly speaking, virtuousness means “excellence” and refers to the pursuit of human flourishing (Cameron et al., 2003; Bright et al., 2006). Bright et al. (2006) made a distinction between *virtuousness in the organization*, which refers to “the behavior of individuals in organizational settings that helps people flourish as human beings” (p. 252) and *virtuousness through organization*, which is the ability of an organization to support virtuous activities among its members (Cameron et al., 2004; Cameron, 2008; Spreitzer et al., 2012) through organizational characteristics such as attributes, structure, systems and processes that promote the optimal functioning of employees (Cameron et al., 2003; Cameron and Caza, 2004; Nikandrou and Tsachouridi, 2015; Sison and Ferrero, 2015). In line with this conceptual framework, the new integrative construct of VOP presented here is defined as formal organizational practices that focus on employees’ psychological well-being and optimal health. VOP differ from commonly defined organizational practices (e.g., *High-performance work system*, *high-commitment work system*, and *Strategic human resources management*) because they prioritize workers’ well-being over their performance goals. The organizational practices included in this new construct were identified through: (1) the APA’s empirical model of *Psychologically healthy workplace*, and (2) an in-depth review of the scientific literature on the organizational practices that are commonly associated with well-being at work.

The APA’s *Psychologically healthy workplace* program has significant international influence. It was initiated in 1999 to recognize and support organizations fostering the health and well-being of their employees through practices, programs and policies that could thus be considered as virtuous practices. This model highlights five “virtuous” organizational practices:

1. Practices of Participative Decision-Making (PPDM), also associated with organizational democracy, refer to “ongoing, broad-based, and institutionalized employee participation that is not *ad hoc* or occasional in nature” (Weber et al., 2020, p. 1009). These practices are numerous and include decision-making meetings, problem-solving groups, formal suggestion systems (e.g., suggestion boxes), referendums, opinion surveys, and worker-supervisor meetings to determine goals and work methods (Lavelle et al., 2010; Wilkinson et al., 2010; Marchington, 2015). Numerous studies have demonstrated that these practices are positively associated with positive emotions, job satisfaction, affective commitment and organizational commitment, and negatively related to negative emotions (e.g., Witt et al., 2000; Kooij et al., 2010; Mendelson et al., 2011; Ogbonnaya et al., 2017). They allow employees to exercise control over their professional environment and offer an essential resource for escaping from stressful situations (Probst, 2005). More precisely, PPDM provide opportunities for workers to gain more information, to better understand organizational processes, be engaged in problem-solving and to influence organizational choices by expressing their thoughts, opinions and ideas and defending their interests (Bogler and Somech, 2005; Somech, 2010). Finally, PPDM show employees that they are valued by their superior and their organization (Yoerger et al., 2015; Weber et al., 2020).
2. Work-Life Balance Practices (WLBP) refer to “discretionary and formal organizational policies, services, and benefits aimed at reducing employees’ work–family conflict and/or supporting their family roles outside the workplace” (Masterson et al., 2021, p. 118). The meta-analysis of 57 studies by Butts et al. (2013) confirmed that WLBP are positively related to job satisfaction, affective commitment and intention to stay, and negatively related to work-family conflict. They enable employees to choose their working hours (e.g., flextime, compressed workweek), working time (e.g., part-time) as well as the work location (e.g., telecommuting; Hill et al., 2008; Bae and Goodman, 2014). They also provide instrumental support through financial assistance, child-care services and elder-care assistance (Bae and Goodman, 2014). They thus help workers to be more flexible in carrying out their work in order to meet their professional and personal requirements (Jahn et al., 2003; Cogin et al., 2017).
3. Health and Safety Practices (HSP) are those that promote employee health and safety within the organization (Mearns et al., 2010; Huang et al., 2013; Parker et al., 2017; Schulz et al., 2017). They differ from the concept of psychosocial safety climate (PSC) in that they consider the overall health (i.e., psychological, physical) of employees and not just their psychological health (Hall et al., 2010). These practices are related positively with job satisfaction, work engagement and well-being and negatively with emotional exhaustion and psychological distress (e.g., Grawitch et al., 2007; Nielsen et al., 2011; Gragnano et al., 2017). Their aim is to create a safe professional environment and to reduce psychosocial risks, accidents and injuries in the workplace (Christian et al., 2009; Dollard and Bakker, 2010; Nahrgang et al., 2011; Dollard et al., 2012). They show employees that management values and prioritizes their well-being over performance goals (Hall et al., 2010). Interestingly, while the APA model considers “Health and Safety” as a single dimension, several authors recommend that they should be studied as two separate constructs (Mearns et al., 2010; Zweber et al., 2016; Parker et al., 2017).
4. Recognition Practices (RP) are those that give employees feedback on their work and reward them for their contribution to the organization and their professional achievements (Fallon and Rice, 2015). RP are numerous, and include bonuses, promotion, congratulations, awards, and improving working conditions (Brun and Dugas, 2005; Fall, 2015). They can be classified according to their purpose, namely: recognition-relationship (i.e., practices showing employees that they are valued as individuals), recognition-reward (i.e., practices recognizing an outcome), and recognition-accomplishment (i.e., practices that give the employee a sense of purpose; Roche, 2014). RP promote the development of self-confidence and self-esteem (El Akremi et al., 2009; Fallon and Rice, 2015) and are positively associated with well-being indicators such as job satisfaction, affective commitment and intrinsic motivation (e.g., Kooij et al., 2010; Fall, 2015). They also have negative relationships with job stress, fatigue, emotional exhaustion, work–life imbalance and turnover intention (e.g., Grawitch et al., 2007; Boxall and Macky, 2014). Some authors advocate combining different types of RP (e.g., feedback on work, monetary recognition, and social recognition), which has been shown to have a greater positive effect on behavior (e.g., task performance) than each reward practice considered in isolation (Stajkovic and Luthans, 2003).
5. Practices of Career Management (PCM), also called “growth and development,” are defined as “programs, processes, and other forms of assistance provided by organizations to support and enhance their employees’ career success” (Kong et al., 2011, p. 112). They include training programs, career counseling, formal mentoring, internal mobility, outplacement, and pre-retirement programs (Bagdadli and Gianecchini, 2019). Numerous studies have demonstrated that PCM are positively related to job satisfaction, affective commitment, well-being and intention to remain (e.g., Armstrong-Stassen and Ursel, 2009; Rego and Cunha, 2009; Kooij et al., 2010) and negatively associated with emotional exhaustion and turnover intention (Grawitch et al., 2007). Indeed, PCM have substantial value for workers by helping them develop their knowledge and skills and use them in new situations (Grawitch et al., 2007), challenging them, stimulating them to expand their potential, and supporting their employability (Armstrong-Stassen and Stassen, 2013). They are also a sign that the organization values them and is prepared to make long-term investment in their career (Paré and Tremblay, 2007). Finally, these practices make it possible to match the employees’ interests, aspirations, and abilities with organizational opportunities.

Finally, communication is the foundation for all *psychologically healthy workplace* practices, by showing workers that the organization recognizes their existence. Also referred to as “sharing information,” Communication Practices (CP) are “a set of practices set up by organizations to disseminate and receive information” (Tremblay et al., 2000, p. 4). Several studies have observed that CP have positive relationships with job satisfaction and affective commitment, and negative relationships with job stress, fatigue and work–life imbalance (e.g., Kooij et al., 2010; Mendelson et al., 2011; Boxall and Macky, 2014). Indeed, CP allow workers to understand what the organization expects of them regarding their role, goals, and tasks (Tremblay et al., 2000; van Vuuren et al., 2007). Furthermore, by receiving the right information at the right time, workers are able to understand and predict organizational plans and can adapt their behavior accordingly (Lages et al., 2005). Finally, the transmission of accurate information promotes the development of a climate of trust and mutual respect (Sousa-Lima et al., 2013). Finally, these practices can also be a signal that the organization cares about the well-being, concerns and opinions of workers (Zhang and Agarwal, 2009).

In brief, this model focuses on the formal practices that organizations can implement and optimize in order to promote their employees’ psychological health. However, it does not include two other organizational practices that have been shown to have positive effects on employees’ health, namely, Organizational Justice Practices (OJP) and Social Dialogue Practices (SDP).

Many scientists have argued that organizational justice is an important factor of workers’ health. More precisely, in the early 2000s, procedural and distributive equity (Wilson et al., 2004) as well as the broader notion of fairness (Kelloway and Day, 2005) were identified as features of “healthy” work environments. More recently, Guest (2017) added equal opportunities and fair rewards in his descriptive model of practices promoting the psychological health of employees. Several meta-analyses have also reported the beneficial nature of organizational justice on employees’ well-being, with positive relationships with job satisfaction, organizational commitment and positive emotions, and negative relationships with negative emotions, withdrawal and counterproductive work behavior (Colquitt et al., 2001, 2013). Moreover, no study has shown any deleterious effect of these practices on the psychological health of employees. OJP show workers that the organization respects them, cares about their well-being, supports them and values them (Le et al., 2018). For this reason, we considered that they should be included in our new VOP construct. OJP include fair compensation (e.g., wages, bonuses, and promotions; Adams, 1965), formalization of compensation criteria (e.g., transparent salary policy, merit pay, communication of criteria for awarding bonuses; Thibaut and Walker, 1975), equitable transmission of information (e.g., information meeting; Greenberg, 1993) as well as fair treatment (e.g., equal opportunity program, program to prevent discrimination at work; Colquitt, 2001).

Similarly, several empirical studies have provided evidence that SDP, also called social collective bargaining, joint consultation or industrial relations climate, are positively associated with indicators of well-being such as job satisfaction, organizational commitment, and work–life balance (e.g., Deery and Iverson, 2005; Conway and Monks, 2008; Snape and Redman, 2012; De Prins et al., 2018). These practices give workers the opportunity to express themselves and protect their interests (Deery et al., 2014). They also improve the clarity of mutual objectives, as well as the working conditions and wages of workers (Deery et al., 1999). Finally, SDP show that the organization listens, considers and supports its employees, in other words, that it promotes organizational trust (Newman et al., 2018). Moreover, like OJP, no study has shown any deleterious effect of SDP on the psychological health of employees. For this reason, Guest (2017) included collective representation in his descriptive model. De Prins et al. (2018) added that the industrial relations climate is an essential factor in protecting the health of employees. Examples of SDP include formal and regular collective bargaining and consultation with employees whenever an organizational change affecting them occurs (Guest, 2017; De Prins et al., 2018).

In sum, the new integrative psychological construct of VOP has eight dimensions: PPDM, work-life balance, health and safety, recognition, career management, communication, organizational justice, and social dialogue.

### The Assessment of Virtuous Organizational Practices

Significant divergences exist among researchers about how to measure organizational practices. The first concerns the source of the data. Initial studies focused on organizational practices from the perspective of HR actors and managers, ignoring how they were experienced by workers (Liao et al., 2009). More recently, differences in the perception of organizational practices in different professional fields (Wang et al., 2020; Beijer et al., 2021; Van Beurden et al., 2021) and the recognition that employees’ attitudes and behaviors are largely determined by their perception of these practices (Edgar and Geare, 2005) have led to a growing body of research based on employee reports. For example, Beijer et al. (2021) observed that only 9% of studies published between 2000 and 2002 used an employee-level rating vs. 91% using a management-level rating, compared to 37% and 63%, respectively, between 2015 and 2017. Following this trend, the VOPi measures the subjective VOP at the employee level.

A second difference concerns objective vs. subjective measures of organizational practices (Mendelson et al., 2011). In their literature reviews, Beijer et al. (2021) and Wang et al. (2020) found that the main difference in the scales evaluating organizational practices lay in whether they used objective (e.g., existence, frequency of use) or subjective (e.g., satisfaction) measure. According to Spector et al. (2019), these two forms of evaluation refer, respectively, to factual and perceptual constructs, the former involving cognitive and quantitative evaluations based on observable and verifiable elements, while the latter involve emotional and qualitative evaluations based on elements requiring a more personal interpretation. However, workers’ health, attitudes and behaviors can be predicted more by the availability of practices than their use (Butts et al., 2013). Moreover, Beijer et al. (2021) observed that objective evaluation involves practices measured by managers, while subjective evaluation refers to practices measured by employees. In line with these studies, VOPi was developed as a subjective measure of VOP as perceived by employees.

A final difference concerns the relations between different organizational practices, their combined effect on the attitudes, behaviors and health status of employees, and consequently the statistical approach adopted in their operational modeling. Initially, the effects of these practices were considered as independent, but there is now broad agreement that they have a synergistic effect (Delery, 1998; Chadwick, 2010; Boon et al., 2019). In other words, the effects of organizational practices are mutually reinforcing, their combined effects being greater than the sum of their independent effects (Chadwick, 2010). Consequently, we hypothesize that VOP could be modeled by a global factor.

## Validation Studies

### Study 1: Initial Development of Virtuous Organizational Practices Inventory

The aim of study 1 was to generate a preliminary pool of items. The deductive approach was used to develop an initial version of VOPi based on relevant theoretical and conceptual knowledge (Boateng et al., 2018), following an in-depth review of the literature and tools of the eight sub-dimensions of the VOPi.

For a short and easy-to-use tool, five items were generated per dimension, except for OJP for which eight items were created due to its multidimensional nature (Colquitt, 2001). Moreover, as previously noted, while HSP are considered as a single dimension in the APA model, many researchers consider that these two constructs are independent (Mearns et al., 2010; Zweber et al., 2016; Parker et al., 2017), and we therefore created five items for each. In total, the scale comprised 48 items; twenty were directly derived from existing scales (41.67%; e.g., “*Mon organization favorise la mobilité interne*” [My organization promotes internal mobility]) and 28 were created by a committee of experts (i.e., The committee of experts included a full professor, a lecturer, a Ph.D. student and a psychologist, all specialized in work and organizational psychology; 58.33%; e.g., “*Dans mon organization, les salarié(e)s participent aux prises de décisions liées aux changements en interne*” [In my organization, employees are involved in making decisions about internal changes]). The items were then randomized to avoid a contamination effect of the responses (El Akremi et al., 2018).

To check the clarity of the items, we performed two pre-tests. The first was carried out with 13 workers from various professional sectors, and the second with 22 experts in work psychology. Participants were asked to rate the degree of clarity of each item on a 7-point Likert scale ranging from 1 (“*The item is not at all clear*”) to 7 (“*The item is very clear*”). They were asked to indicate items that were unclear by adding comments. This allowed us to identify alternative wording for items that had the lowest levels of clarity. Following their comments, we slightly reworded 11 items.

### Study 2: Exploratory Factor Analysis

The aim of study 2 was to explore the factorial structure and psychometric qualities of the VOPi.

#### Method

The Tours-Poitiers Ethics Committee for Research, which is part of the University of Tours (France), approved this research (CER-TP n° 2019-03-02). Participants were informed of the voluntary, anonymous and confidential nature of the study and were asked to give their informed consent. This study was thus conducted in accordance with the Helsinki Declaration (World Medical Association, 2013).

##### Participants

A total of 606 French workers (168 men, 437 women, and 1 non-respondent) from various sectors of activity participated in this study; 256 worked in private organizations (42.24%), 234 in non-profit organizations (38.61%), and 116 in public organizations (19.14%). Their average age was 36.78 years (*SD* = 10.86, range 18 to 69), their average job tenure was 6.36 years (*SD* = 7.19) and their average organizational tenure was 7.96 years (*SD* = 8.16). Among the participants, 503 worked full-time (83.00%), 102 part-time (16.83%) and 1 did not provide this information; 522 participants were in permanent work (86.14%); and 84 in temporary work (13.86%).

##### Measure

Participants were instructed to complete the preliminary 48-item version of the VOPi as follows: “*The following items are about the practices of your organization. Indicate your degree of agreement with each one.*” They responded on a five-point Likert scale ranging from 1 (“*Strongly disagree*”) to 5 (“*Strongly agree*”).

#### Statistical Analysis and Results

In accordance with the recommendations of Tabachnick and Fidell (2013), we carried out preliminary analyses, which revealed no univariate outliers (i.e., |*z*| > 3.29, *p* < 0.001). The kurtosis and skewness coefficients were all acceptable, with values less than 10 for all components (George and Mallery, 2010; Kline, 2016). The mean score for all items was 3.10 out of 5 (Minimum = 2.20; Maximum = 3.78).

We then carried out an Exploratory Factor Analysis (EFA) using SPSS version 25. To determine the number of factors to retain, we used the most frequently used criteria, namely the Kaiser-Guttman criterion (Kaiser, 1960), the scree plot (Cattell, 1966), the proportion of variance explained (Boateng et al., 2018), and theoretical interpretability (Conway and Huffcutt, 2003). In addition, because significant and positive relationships have been found between some VOP (Simard et al., 2005; Edgar and Geare, 2009; Ogbonnaya et al., 2017; Loh et al., 2018), we used oblique rotation (Tabachnick and Fidell, 2013). In this way, we identified an eight-factor solution whose eigenvalues were all greater than 1 and explained 61.90% of the total variance of the 48 items. Items that showed cross-loading were then removed (Boateng et al., 2018). Moreover, to make VOPi as parsimonious as possible, and because researchers are increasingly creating short scales with an average of three items per dimension (Moneta, 2017; Haar et al., 2019; Décieux et al., 2020), we retained only the three items with the highest loadings per dimension. We made sure that these were representative of the measured constructs and verified the absence of redundancy in the formulation of the retained items.

A second EFA was then carried out, again using oblique rotation. A four-factor solution explaining 58.45% of the total variance of the 24 items was identified. However, this was not theoretically interpretable. We therefore performed a final EFA, fixing the theoretically expected number of factors (i.e., eight). Using the criteria described above, we retained a final eight-factor solution explaining 72.45% of the total variance with 24 items. In accordance with the recommendations of Tabachnick and Fidell (2013) and Boateng et al. (2018), no item had saturations less than 0.32 (Table 1). The internal consistency of each factor was satisfactory (Nunnally, 1978) with values ranging from 0.72 to 0.84 (Table 1). These eight factors also represented a factorial solution that was easily interpretable. An examination of the interpretability of the factors showed that the first factor corresponded to the PPDM dimension, the second to WLBP, the third to HSP, the fourth to RP, the fifth to PCM, the sixth to CP, the seventh to OJP, and the eighth to SDP. (VOPi can be provided by the first author on request). Descriptive statistics of the 24 items are presented in Table 1.

**TABLE 1:** Means, standard deviations, and factor loadings of the exploratory factor analysis (Study 2).

Items	*M*	SD	Skewness	Kurtosis	PPDM	WLBP	HSP	RP	PCM	CP	OJP	SDP
Item 1	2.39	1.12	0.50	–0.60	**0.82**	0.01	0.01	0.02	0.01	0.04	0.07	0.01
Item 2	2.40	1.07	0.40	–0.61	**0.81**	0.02	0.04	0.00	0.04	0.05	0.02	0.02
Item 3	2.57	1.07	0.12	–0.85	**0.54**	0.03	0.03	0.08	0.04	0.10	0.01	0.13
Item 4	2.88	1.23	–0.05	–1.07	0.06	**0.86**	0.07	0.02	0.10	0.09	0.02	0.06
Item 5	2.20	1.27	0.72	–0.65	0.03	**0.56**	0.08	0.04	0.07	0.08	0.09	0.05
Item 6	3.20	1.23	–0.44	–0.89	0.04	**0.52**	0.03	0.17	0.07	0.04	0.08	0.06
Item 7	3.32	1.01	–0.56	0.08	0.02	0.03	**0.49**	0.01	0.18	0.06	0.11	0.14
Item 8	3.03	1.08	–0.24	–0.73	0.06	0.06	**0.72**	0.04	0.05	0.08	0.02	0.04
Item 9	3.17	1.03	–0.34	–0.49	0.08	0.06	**0.75**	0.04	0.00	0.05	0.03	0.00
Item 10	3.25	1.15	–0.38	–0.71	0.19	0.02	0.12	**0.52**	0.03	0.16	0.18	0.02
Item 11	3.25	1.06	–0.44	–0.56	0.04	0.07	0.05	**0.41**	0.06	0.18	0.28	0.06
Item 12	2.98	1.08	–0.26	–0.75	0.04	0.10	0.03	**0.52**	0.13	0.04	0.05	0.13
Item 13	3.45	1.13	–0.52	–0.50	0.08	0.05	0.16	0.20	**0.37**	0.05	0.09	0.05
Item 14	3.27	1.06	–0.46	–0.46	0.07	0.02	0.05	0.24	**0.49**	0.18	0.07	0.00
Item 15	3.35	1,00	–0.42	–0.24	0.02	0.13	0.02	0.05	**0.44**	0.08	0.04	0.09
Item 16	3.43	1.04	–0.79	–0.01	0.08	0.06	0.07	0.06	0.01	**0.58**	0.08	0.03
Item 17	3.48	1.01	–0.86	0.17	0.08	0.03	0.08	0.12	0.18	**0.58**	0.12	0.04
Item 18	3.33	1.08	–0.61	–0.34	0.15	0.12	0.11	0.13	0.18	**0.33**	0.06	0.09
Item 19	2.95	1.22	–0.04	–1.11	0.18	0.07	0.08	0.02	0.03	0.01	**0.65**	0.02
Item 20	3.18	0.98	–0.37	–0.27	0.11	0.02	0.09	0.03	0.06	0.01	**0.59**	0.05
Item 21	3.78	1.14	–0.93	0.15	0.07	0.01	0.05	0.06	0.00	0.11	**0.48**	0.05
Item 22	3.17	0.91	–0.40	0.19	0.06	0.02	0.08	0.02	0.06	0.07	0.03	**0.80**
Item 23	3.28	0.97	–0.65	0.06	0.02	0.02	0.27	0.01	0.06	0.09	0.01	**0.55**
Item 24	3.09	0.94	–0.43	–0.05	0.01	0.03	0.13	0.02	0.09	0.11	0.06	**0.60**
α					0.84	0.72	0.82	0.81	0.74	0.78	0.74	0.81

*PPDM, Practices of Participative Decision-Making; WLBP, Work-Life Balance Practices; HSP, Health and Safety Practices; RP, Recognition Practices; PCM, Practices of Career Management; CP, Communication Practices; OJP, Organizational Justice Practices; and SDP, Social Dialogue Practices. The bold values correspond to the items saturations selected for each dimension.*

Inter-item correlations are presented in Table 2 and inter-factor correlations in Table 3.

**TABLE 2:** Inter-item correlations (Study 2).

	1	2	3	4	5	6	7	8	9	10	11	12	13	14	15	16	17	18	19	20	21	22	23	24
Item 1	1	0.70	0.58	0.33	0.24	0.31	0.30	0.40	0.41	0.50	0.45	0.41	0.36	0.42	0.26	0.38	0.37	0.43	0.48	0.45	0.28	0.42	0.39	0.39
Item 2		1	0.60	0.38	0.22	0.39	0.33	0.43	0.44	0.46	0.48	0.44	0.42	0.45	0.28	0.44	0.42	0.48	0.45	0.48	0.27	0.40	0.43	0.40
Item 3			1	0.37	0.20	0.30	0.33	0.45	0.42	0.46	0.46	0.44	0.37	0.42	0.27	0.44	0.37	0.45	0.41	0.42	0.30	0.42	0.44	0.43
Item 4				1	0.48	0.53	0.17	0.34	0.31	0.29	0.35	0.32	0.24	0.31	0.27	0.35	0.25	0.37	0.31	0.29	0.18	0.31	0.30	0.30
Item 5					1	0.36	0.21	0.26	0.26	0.24	0.24	0.27	0.19	0.24	0.23	0.18	0.18	0.28	0.22	0.26	0.17	0.18	0.21	0.19
Item 6						1	0.26	0.33	0.36	0.34	0.36	0.40	0.29	0.38	0.28	0.32	0.27	0.38	0.28	0.28	0.22	0.30	0.31	0.34
Item 7							1	0.54	0.54	0.38	0.33	0.40	0.39	0.44	0.36	0.35	0.38	0.39	0.30	0.40	0.35	0.40	0.49	0.41
Item 8								1	0.71	0.45	0.49	0.45	0.47	0.50	0.33	0.47	0.47	0.51	0.37	0.46	0.30	0.42	0.60	0.51
Item 9									1	0.42	0.46	0.45	0.49	0.50	0.35	0.47	0.45	0.50	0.34	0.43	0.30	0.40	0.58	0.48
Item 10										1	0.58	0.60	0.46	0.48	0.26	0.33	0.27	0.37	0.49	0.49	0.34	0.38	0.41	0.37
Item 11											1	0.57	0.45	0.52	0.28	0.46	0.42	0.45	0.50	0.54	0.40	0.41	0.47	0.43
Item 12												1	0.43	0.51	0.34	0.40	0.34	0.52	0.42	0.47	0.32	0.42	0.45	0.42
Item 13													1	0.62	0.37	0.39	0.37	0.46	0.39	0.43	0.33	0.40	0.45	0.38
Item 14														1	0.44	0.48	0.50	0.52	0.43	0.50	0.32	0.40	0.49	0.40
Item 15															1	0.33	0.39	0.36	0.29	0.31	0.20	0.32	0.37	0.28
Item 16																1	0.59	0.52	0.37	0.38	0.35	0.36	0.45	0.42
Item 17																	1	0.52	0.34	0.40	0.30	0.32	0.48	0.43
Item 18																		1	0.35	0.39	0.36	0.42	0.49	0.44
Item 19																			1	0.60	0.43	0.36	0.37	0.36
Item 20																				1	0.44	0.41	0.42	0.43
Item 21																					1	0.29	0.30	0.30
Item 22																						1	0.60	0.58
Item 23																							1	0.61
Item 24																								1

*All correlations are significant at *p* < 0.01.*

**TABLE 3:** Inter-factor correlations (Study 2).

		1	2	3	4	5	6	7	8
1	PPDM	1	0.44	0.52	0.62	0.52	0.58	0.56	0.56
2	WLBP		1	0.41	0.46	0.40	0.43	0.38	0.39
3	HSP			1	0.60	0.61	0.63	0.54	0.56
4	RP				1	0.59	0.56	0.63	0.57
5	PCM					1	0.62	0.51	0.65
6	CP						1	0.53	0.60
7	OJP							1	0.51
8	SDP								1

*All correlations are significant at *p* < 0.01. Note. PPDM, Practices of Participative Decision-Making; WLBP, Work-Life Balance Practices; HSP, Health and Safety Practices; RP, Recognition Practices; PCM, Practices of Career Management; CP, Communication Practices; OJP, Organizational Justice Practices; and SDP, Social Dialogue Practices.*

### Study 3: Confirmatory Factor Analysis

The purpose of Study 3 was to assess the factorial structure of the 24-item version of the VOPi identified in Study 2, through confirmatory factor analysis with a new sample. We compared alternative models to identify the best representation of the data (i.e., second-order factor model, single first-order factor, and eight first-order factors).

#### Method

The third study was also conducted in accordance with the Helsinki Declaration (World Medical Association, 2013), guaranteeing the anonymity, informed consent and absolute confidentiality of participants. This study was also approved by the Tours-Poitiers Ethics Committee for Research (CER-TP, n° 2019-03-02).

##### Participants

The sample consisted of 483 French workers, including 136 men (28.16%), and 347 women (71.84%). They had an average age of 39.10 years (*SD* = 11.58, range 18 to 67). Their average job tenure was 5.25 years (*SD* = 6.29) and their average organizational tenure was 8.00 years (*SD* = 8.74); 398 worked full-time (82.40%) and 85 part-time (17.60%), and 382 participants were in permanent work (79.09%) and 101 in temporary work (20.91%). Finally, 232 worked in private organizations (48.03%), 130 in public organizations (26.92%), 119 in non-profit organizations (24.64%), and 2 did not provide this information.

##### Measure

Participants completed the 24-item version of the VOPi, with the same instruction: “*The following items are about the practices of your organization. Indicate your degree of agreement with each one.*” They responded using a five-point Likert scale ranging from 1 (“Strongly disagree”) to 5 (“Strongly agree”).

#### Statistical Analysis and Results

No univariate outliers were identified (i.e., |*z*| > 3.29, *p* < 0.001; Tabachnick and Fidell, 2013). However, and in accordance with Tabachnick and Fidell’s recommendations (2013), six multivariate outliers [i.e., Mahalanobis distance greater than χ^2^(8) = 26.12, *p* < 0.001] were excluded, leaving 477 participants for analyses. The kurtosis and skewness coefficients were all adequate with values less than 10 for all components (George and Mallery, 2010; Kline, 2016). All components were therefore considered to be normally distributed. The average score for all items was 3.10 out of 5 (Minimum = 2.37; Maximum = 3.62). Descriptive statistics for each dimension are presented in Table 4 and correlations between dimensions in Table 5.

**TABLE 4:** Descriptive statistics of the eight subdimensions of VOPi (Study 3).

		Min	Max	M	SD	Skewness	Kurtosis	α
1	PPDM	1	5	2.52	0.96	0.28	–0.40	0.84
2	WLBP	1	5	2.86	1.04	–0.03	–0.74	0.73
3	HSP	1	5	3.19	0.99	–0.40	–0.42	0.83
4	RP	1	5	3.11	1,00	–0.17	–0.67	0.85
5	PCM	1	5	3.31	0.89	–0.51	–0.13	0.76
6	CP	1	5	3.29	0.90	–0.46	–0.06	0.77
7	OJP	1	5	3.17	0.94	–0.31	–0.45	0.74
8	SDP	1	5	3.02	0.87	–0.26	–0.05	0.82

*PPDM, Practices of Participative Decision-Making; WLBP, Work-Life Balance Practices; HSP, Health and Safety Practices; RP, Recognition Practices; PCM, Practices of Career Management; CP, Communication Practices; OJP, Organizational Justice Practices; and SDP, Social Dialogue Practices.*

**TABLE 5:** Correlations between dimensions of VOPi (Study 3).

		1	2	3	4	5	6	7	8
1	PPDM	1	0.45	0.55	0.69	0.54	0.61	0.57	0.70
2	WLBP		1	0.47	0.56	0.48	0.43	0.35	0.49
3	HSP			1	0.64	0.65	0.61	0.50	0.67
4	RP				1	0.61	0.64	0.70	0.69
5	PCM					1	0.58	0.53	0.58
6	CP						1	0.62	0.60
7	OJP							1	0.57
8	SDP								1

*PPDM, Practices of Participative Decision-Making; WLBP, Work-Life Balance Practices; HSP, Health and Safety Practices; RP, Recognition Practices; PCM, Practices of Career Management; CP, Communication Practices; OJP, Organizational Justice Practices; and SDP, Social Dialogue Practices.*

Next, we performed a confirmatory factor analysis with maximum likelihood estimation method using AMOS software Version 25. We used the following fit indices: chi-square (Jöreskog, 1967), standardized root mean square residual (SRMR; Jöreskog and Sörbom, 2001; ≤0.05 good fit and ≤0.10 acceptable fit), root mean square error of approximation (RMSEA; Browne and Cudeck, 1989; ≤0.05 good fit and ≤0.08 acceptable fit), comparative fit index (CFI; Bentler, 1990; ≥0.90 acceptable fit), Akaike information criterion (AIC; Akaike, 1987), and χ^2^/df (Kline, 2016).

**FIGURE 1:**
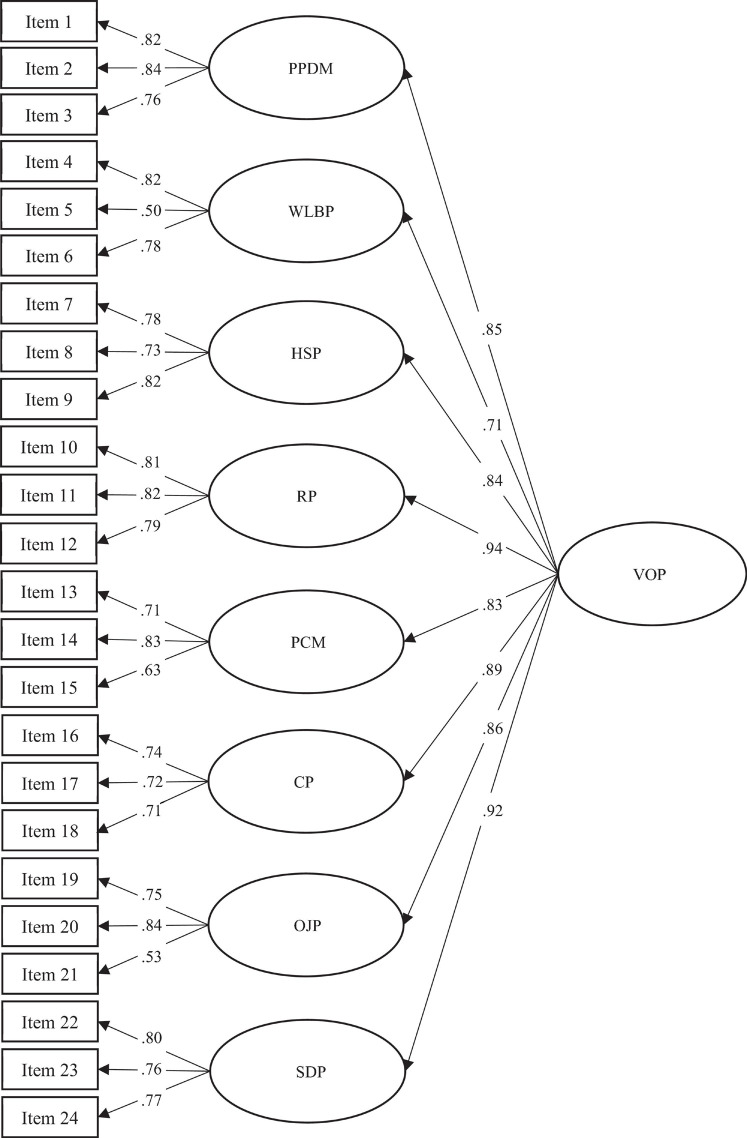
CFA with second-order factor model (Study 3). All correlations are significant at *p* < 0.01.

We tested a series of models. The first was a second-order factor model with eight first-order factors. The results demonstrated a good fit of the theoretical model to the data [χ^2^ = 584.76 (244), *p* < 0.001; SRMR = 0.04; RMSEA = 0.05; CFI = 0.95; AIC = 744.76; χ^2^/df = 2.40] (Figure 1). The second model included only a single first-order factor; the fit of the theoretical model to the data was not satisfactory [χ^2^ = 1255.54 (252), *p* < 0.001; SRMR = 0.06; RMSEA = 0.09; CFI = 0.84; AIC = 1399.5; χ^2^/df = 4.98]. Finally, the third model included eight first-order factors; results indicated a good fit of the model to the data [χ^2^ = 469.52 (224), *p* < 0.001; SRMR = 0.03; RMSEA = 0.05; CFI = 0.96; AIC = 669.52; χ^2^/df = 2.10]. Comparison of the second-order factor model with eight first-order factors on the one hand and the eight first-order factor model on the other revealed that the latter fit the data better than the former [Δχ^2^(20) = 115.24, *p* < 0.001]. However, as mentioned above, organizational practices have been shown to have a synergistic, mutually reinforcing effect, and their combination has more influence on the health, attitudes and behaviors of workers than the simple sum of their independent effects (Boon et al., 2019). For this reason, we retained the second-order model.

### Study 4: Virtuous Organizational Practices Inventory Convergent, Predictive and Incremental Validities

The aim of study 4 was to examine the convergent, predictive and incremental validities of the VOPi with a new sample. PSC was used to test the convergent validity (i.e., additional predictive validity over other constructs) of the new scale because it is conceptually similar to the VOP, defined as “policies, practices and procedures for the protection of worker psychological health and safety” (Dollard and Bakker, 2010, p. 579). We then investigated the predictive validity of VOPi through job satisfaction and affective commitment. Job satisfaction refers to “a pleasurable or positive emotional state resulting from the appraisal of one’s job or job experiences” (Locke, 1976, p. 1304), and affective commitment is defined as “employee’s emotional attachment to, identification with, and involvement in the organization” (Meyer and Allen, 1991, p. 67). Several meta-analyses have observed positive relationships between some organizational practices (e.g., internal promotion, training, rewards, participation, work-life policies, and organizational justice) and job satisfaction on the one hand and affective commitment on the other (e.g., Meyer et al., 2002; Kooij et al., 2010; Butts et al., 2013; Colquitt et al., 2013). The final aim was to investigate the incremental validity of the VOPi on job satisfaction and affective commitment beyond the PSC.

#### Method

As for Studies 2 and 3, this last study was also approved by Tours-Poitiers Ethics Committee for Research (CER-TP, n° 2019-03-02) and respected the Helsinki Declaration on research involving human subjects (World Medical Association, 2013).

##### Participants

The sample comprised 283 participants, with 105 (37.10%) men and 178 women (62.90%). Ages ranged from 18 to 67 years, with an average of 33.75 years (*SD* = 10.69), average job tenure of 6.31 years (*SD* = 8.39) and average organizational tenure of 7.34 years (*SD* = 10.41); 238 participants worked full-time (84.10%) and 45 part-time (15.90%), 216 participants were in permanent work (76.33%), and 67 in temporary work (23.67%); 185 worked in the private sector (65.37%), 84 in the public sector (29.68%), and 14 in the non-profit sector (4.95%).

##### Measure

Participants completed the four scales (i.e., VOPi, PSC, job satisfaction, and affective commitment) using a five-point Likert scale ranging from 1 (“*Strongly disagree*”) to 5 (“*Strongly agree*”).

Virtuous organizational practices were assessed with the 24-item version of the VOPi. Analyses revealed good internal consistency for the total inventory (α = 0.94).

*Psychosocial safety climate* was measured with Hall et al.’s (2010) composed of 12 items and four sub-dimensions: 3 items for management commitment (e.g., “Senior management show support for stress prevention through involvement and commitment”), 3 items for management priority (e.g., “Senior management considers employee psychological health to be as important as productivity”), 3 items for organizational communication (e.g., “There is good communication here about psychological safety issues which affect me”) and 3 items for organizational participation (e.g., “Participation and consultation in psychological health and safety occurs with employees’, unions’ and health and safety representatives in my workplace”). The alpha for the global scale was 0.95.

*Affective commitment* was measured with Meyer et al.’s (1993) composed of 6 items (e.g., “This organization has a great deal of personal meaning for me”). The internal consistency of this scale was acceptable (α = 0.77).

*Job satisfaction* was measured using the single item of Tavani et al. (2014; i.e., “Overall, I am satisfied with my work”).

#### Statistical Analysis and Results

To examine convergent and predictive validity, we performed bivariate Pearson correlations between VOPi, PSC, affective commitment, and job satisfaction (Table 6).

**TABLE 6:** Convergent and predictive validity (Study 4).

	1	2	3	4
1. VOPi	1	0.78	0.53	0.60
2. PSC		1	0.54	0.57
3. AC			1	0.54
4. JS				1

*All correlations are significant at *p* < 0.001. Note. VOPi, Virtuous Organizational Practices inventory; PSC, Psychosocial Safety Climate; AC, Affective Commitment; and JS, Job Satisfaction.*

The results for convergent validity demonstrated significantly positive correlations between VOP and PSC (*r* = 0.78, *p* < 0.001). The results for predictive validity indicated significant and positive correlations between VOP on the one hand and affective commitment (*r* = 0.53, *p* < 0.001) and job satisfaction (*r* = 0.60, *p* < 0.001) on the other.

Finally, because the VOPi showed a strong, significant, and positive correlation with PSC (*r* = 0.78, *p* < 0.001), we tested its incremental validity to predict job satisfaction and affective commitment using hierarchical regression analyses. The control variables of gender, age and education level were introduced in step 1. PSC was then introduced in step 2. Finally, VOPi was introduced in step 3. Analyses revealed that the variance inflation factors (VIF) were all less than 10, demonstrating the absence of significant multi-collinearities (Kline, 2016). Results showed that the VOPi added specific variance beyond PSC to explain job satisfaction on the one hand (adjusted Δ*R*^2^ = 0.047, *p* < 0.001; Table 7) and affective commitment on the other (adjusted Δ*R*^2^ = 0.019, *p* < 0.01; Table 8). The incremental validity of the VOPi beyond the PSC is thus clearly demonstrated for job satisfaction and affective commitment.

**TABLE 7:** Incremental validity of VOPi on job satisfaction.

	Job satisfaction						
	Δ adjusted *R*^2^	Δ *F*	*p* (Δ *F*)	β	*t*	*p*	VIF
Step 1	0.015	2.479	0.061				
Gender				–0.135	–2.201	0.029	1.074
Age				–0.079	–1.304	0.193	1.044
Education level				0.036	0.586	0.558	1.098
Step 2	0.298	121.978	< 0.001				
Gender				–0.039	–0.752	0.452	1.105
Age				–0.012	–0.234	0.815	1.059
Education level				0.039	0.745	0.457	1.098
PSC				0.558	11.044	0.000	1.049
Step 3	0.047	21.373	< 0.001				
Gender				–0.024	–0.485	0.628	1.109
Age				0.000	0.009	0.993	1.062
Education level				0.012	0.241	0.810	1.113
PSC				0.267	3.358	0.001	2.794
VOPi				0.369	4.623	< 0.001	2.810

*Δ adjusted *R*^2^, variation of adjusted *R* Square; ΔF, variation of *F*; *p* (Δ*F*), significance of the variation of *F*; β, standardized beta coefficient; and VIF, variance inflation factor.*

**TABLE 8:** Incremental validity of VOPi on affective commitment.

	Affective commitment						
	Δ adjusted *R*^2^	Δ *F*	*p* (Δ *F*)	β	*t*	*p*	VIF
Step 1	–0.008	0.293	0.830				
Gender				–0.047	–0.758	0.449	1.074
Age				0.010	0.170	0.865	1.044
Education level				0.045	0.725	0.469	1.098
Step 2	0.303	121.218	< 0.001				
Gender				0.050	0.945	0.345	1.105
Age				0.078	1.514	0.131	1.059
Education level				0.048	0.911	0.363	1.098
PSC				0.564	11.010	0.000	1.049
Step 3	0.019	8.762	0.003				
Gender				0.059	1.144	0.253	1.109
Age				0.086	1.694	0.091	1.062
Education level				0.030	0.581	0.562	1.113
PSC				0.371	4.501	0.000	2.794
VOPi				0.245	2.960	0.003	2.810

*Δ adjusted *R*^2^, variation of adjusted R Square; Δ*F*, variation of *F*; *p* (Δ*F*), significance of the variation of *F*; β, standardized beta coefficient; and VIF, variance inflation factor.*

## General Discussion

As underlined at the beginning of this paper, studies of organizational practices that promote workers’ psychological health have received less attention than those that are thought to contribute directly to increasing employees’ performance. Moreover, these studies have only examined isolated effects and have not used an integrative conceptual framework. To fill this gap in the scientific literature and develop a more accurate measure, we therefore took an innovative conceptual approach to develop and validate a new scale of organizational practices that foster the psychological health of employees.

More precisely, this paper presents a new multifaceted construct of VOP, based on the conceptual framework of organizational virtuousness (Meyer, 2018). As these organizational practices have been widely demonstrated to promote well-being (Kooij et al., 2010; Butts et al., 2013; Colquitt et al., 2013), they can be defined as “virtuous,” that is to say contributing to the optimal psychological functioning of workers (Bright et al., 2006). Based on the APA’s descriptive model of a *Psychologically Healthy Workplace*, coupled with a review of the literature on practices related to workers’ psychological health, we identified eight VOP: (1) participation in decision-making, (2) work-life balance, (3) health and safety, (4) recognition, (5) career management, (6) communication, (7) organizational justice, and (8) social dialogue. The first six practices were directly derived from the APA model and the last two from the literature review. The VOP is thus a well-defined integrative construct of formal organizational practices that focus on employees’ psychological well-being and optimal health.

The second aim of the present research was to validate a new scale to assess these perceived VOP. Four studies were conducted to develop and test the VOPi in samples of French workers. The goal of Study 1 was to create a pool of 48 items reflecting the eight sub-dimensions of VOP. In study 2, EFA identified a 24-item solution corresponding to the theoretically relevant sub-dimensions of VOP, explaining 72.45% of the total variance. While some authors recommend studying HSP as two independent constructs (Mearns et al., 2010; Zweber et al., 2016; Parker et al., 2017), in the present study, the items assessing these practices loaded on the same factor, validating the “health and safety” dimension in the APA model. Study 3 confirmed the satisfactory psychometric qualities of the VOPi with a second-order factor model. More precisely, analysis revealed that the eight first-order factor model fit the data better than the second-order factor model. However, the latter was consistent with the concept of synergy frequently mentioned in the research field of organizational practices (Chadwick, 2010; Boon et al., 2019). Indeed, the scientific literature shows that the effectiveness of one practice in an organization depends on other practices (Delery, 1998), and that when they are coherent, they have synergistic effects (Chadwick, 2010; Boon et al., 2019). In other words, their combined effects have a greater weight on the attitudes and behaviors of professionals than the sum of their independent effects. Due to this synergistic effect, organizational practices are regularly referred to as a “system” (e.g., High Performance Work System; High Involvement Work System). As observed by Boon et al. (2019) in their recent literature review: “Over the past three decades, a shared consensus has developed that the focus should be on HR systems rather than individual HR practices because the effects of HR practices are likely to depend on the other practices within the system” (p. 2498). We therefore retained the second-order model, as it illustrates the complex links between different organizational practices. Finally, Study 4 confirmed the convergent validity (i.e., with psychological safety climate), the predictive validity (i.e., job satisfaction, affective commitment) and incremental validity of the VOPi in relation to the PSC to predict workers’ job satisfaction and affective commitment. The predictive and incremental validity of the VOPi demonstrates that our integrative construct provides a better understanding of the combined effects of different VOP on employees’ well-being at work. Moreover, the scrupulous respect of the procedure for creating and validating this tool overcomes the limitations observed by Robinson (2018) and Beijer et al. (2021) in their literature reviews of the development of scales in the field of human resources. The deliberately short format of the VOPi also significantly reduces its administration time, despite the number of dimensions measured, so that it can be easily combined with other tools in future research and administered several times without major difficulty.

Although the development and psychometric validation of the VOPi followed a rigorous procedure using several samples of professionals, certain limitations should be mentioned. First, the samples for the second, third and fourth studies were predominantly female. Although findings are inconsistent, several studies have shown that gender moderates the influence of organizational practices on certain employee attitudes and behaviors (Qiao et al., 2009; Andersén and Andersén, 2019; Shin et al., 2020). Future research should thus test the gender invariance of VOPi (Fouquereau et al., 2018) in order to “determine if items used in survey-type instruments mean the same things to members of different groups” (Cheung and Rensvold, 2002, p. 233). Secondly, the VOPi was validated in a French context. However, according to Rabl et al. (2014), cultural differences could influence the organizational practices used in different countries. Therefore, it would also be interesting to test the cultural invariance of our new tool (Zhou et al., 2019). The validation process could also be extended, notably by conducting a test-retest measure to verify the consistency of workers’ perceptions of VOP over time (Cohen and Swerdlik, 2017) and a test of the social desirability of the scale (King and Bruner, 2000). Thirdly, it would be interesting to test the structure of the VOPi in other samples using bifactor exploratory structural equation modeling, a statistical approach increasingly used by the scientific community (Fadda et al., 2020; Gu et al., 2020) as it enables the variance explained by a global factor (e.g., VOP) to be examined simultaneously with the variance explained by specific factors (e.g., eight sub-dimensions), which is not possible with a hierarchical model (Morin et al., 2016). Adopting such a method based on data collection would support our innovative conceptualization and operationalization of VOP. Fourthly, we kept a second-order factor that does not allow us to understand the effects of different combinations of VOP. It would be interesting in a future study to use Latent Profile Analysis to identify various VOP profiles and their more or less favorable effects on the psychological health of employees. Finally, while the predictive validity of the VOPi on job satisfaction and organizational commitment was demonstrated, it would be interesting to test it on other indicators of well-being. Indeed, because VOP promote the psychological health of workers, their relations with flow at work (Gu et al., 2020), flourishing (Diener et al., 2010), or optimal psychological functioning (Jaotombo, 2019) could further confirm their virtuous nature.

Although the validation process should be continued in future research, we hope that the VOPi will contribute to a better understanding of organizational practices favorable to the health of employees, and more broadly to other positive effects. In line with previous studies that have demonstrated relationships between employee well-being on the one hand and individual and organizational performance on the other (Judge et al., 2001; Ford et al., 2011), our results suggest avenues for future research from the perspective of mutual gains (Van De Voorde et al., 2012), whereby employees and employers both benefit from organizational practices that foster employee well-being, leading in turn to improved operational and financial performance. In other words, organizations that implement VOP protect workers’ psychological health, which in turn contributes indirectly to the organization’s effectiveness.

From an applied perspective, the construct of VOP and its associated inventory (VOPi) are also interesting and useful for consultants and managers. First, because VOP are designed at the strategic level by the employer (Wright and Nishii, 2006) and are organizational determinants of the work environment (Bakker and Demerouti, 2007; Hobfoll, 2011), their implementation and optimization are related to primary prevention (i.e., prevention of disease before it occurs). However, although authors agree that primary prevention is significantly more effective than secondary and tertiary prevention in preserving the health of professionals (Rouat et al., 2017), they also observe that it is less common (Hansez et al., 2009). VOP therefore represent a new, innovative and relevant framework for interventions in the field of psychological health at work.

Secondly, the VOPi complements the set of tools used by professionals to diagnose and support work organizations. It is a short and reliable scale to assess perceived VOP and their effect on workers’ psychological health and attitudes (i.e., job satisfaction, affective commitment), going beyond existing tools, such as the PSC scale (Hall et al., 2010).

Moreover, the multidimensional structure of the VOPi offers the possibility of using the whole or only some dimensions of the inventory. It also allows professionals and organizations to diagnose and compare the use of VOP. It can be used to identify: (1) the practices to be capitalized on to promote the quality of life at work of employees because the analyses show that they had the strongest positive relationships with the employees’ health, attitudes and positive behaviors, and (2) the practices perceived as being under-used and requiring optimization. Use of the VOPi in an organization and identifying the organizational practices to be optimized would thus make it possible to recommend actions to be set up for each type of practice. For example, PPDM could be promoted by scheduling consultation or problem-solving groups, allowing employees to express their opinions on specific issues and thus contribute to collective decisions. WLBP could be optimized by giving employees opportunities to organize their work flexibly in order to meet the demands of their different life roles (e.g., flexible hours, part-time work, and telecommuting), or giving them tangible support (e.g., financial assistance and child-care facilities). Finally, VOPi could also be used to compare departments within the same organization, organizations in the same group, or groups in the same sector, in order to identify professional environments where VOP should be optimized as a priority.

## Conclusion

In conclusion, we hope that the new meta-construct of VOP will contribute to the conceptual clarification of organizational practices favorable to the psychological health of employees and to stimulating research in this field by providing a new statistically valid inventory (VOPi) filling a gap in the literature and in the promotion of psychological health at work.

## Data Availability Statement

The raw data supporting the conclusions of this article will be made available by the authors, without undue reservation. VOPi can be provided by the corresponding author on request.

## Ethics Statement

The studies involving human participants were reviewed and approved by the Tours-Poitiers Ethics Committee for Research (CER-TP, n_ 2019-03-02). Written informed consent for participation was required for this study in accordance with the national legislation and the institutional requirements.

## Author Contributions

JA-B, EF, and SC contributed to the conception and design of the study, and wrote the first draft of the manuscript. JA-B and FL carried out the data collection. HC and JA-B organized the database and performed the statistical analyses. All authors contributed to manuscript revision, read, and approved the submitted version.

## Conflict of Interest

FL headed AD Conseil. The remaining authors declare that the research was conducted in the absence of any business or financial relationship that could be construed as a potential conflict of interest.

## Publisher’s Note

All claims expressed in this article are solely those of the authors and do not necessarily represent those of their affiliated organizations, or those of the publisher, the editors and the reviewers. Any product that may be evaluated in this article, or claim that may be made by its manufacturer, is not guaranteed or endorsed by the publisher.
